# A Dentist Fined for Giving Chloroform

**Published:** 1873-09

**Authors:** 


### A DENTIST FINED FOR GIVING CHLOROFORM.

(*The Clinic;* from the *France Me'dicale*, April 23, 1873).—M. Debaralle, dentist at Lille, was brought up before the *audience correctionelle* for having given chloroform without the diploma of doctor in medicine or officer of health, and for having through "*imprudence, negligence, maladresse et inobservation des reglements*" committed an involuntary homicide by chloroform upon the person of a woman named Caron.

The tribune decided that the administration of chloroform without a diploma was an illegal practice of medicine, and that the defendant was guilty of homicide. As he was guilty of two offenses, he was fined 15 francs for the first, and for the second 500 francs, with imprisonment for one month.

